# *Trans*-pairing between osteoclasts and osteoblasts shapes the cranial base during development

**DOI:** 10.1038/s41598-018-38471-w

**Published:** 2019-02-13

**Authors:** Mio Edamoto, Yukiko Kuroda, Masaki Yoda, Katsuhiro Kawaai, Koichi Matsuo

**Affiliations:** Laboratory of Cell and Tissue Biology, Keio University School of Medicine, Tokyo, 160-8582 Japan

## Abstract

Bone growth is linked to expansion of nearby organs, as is the case for the cranial base and the brain. Here, we focused on development of the mouse clivus, a sloping surface of the basioccipital bone, to define mechanisms underlying morphological changes in bone in response to brain enlargement. Histological analysis indicated that both endocranial and ectocranial cortical bone layers in the basioccipital carry the osteoclast surface dorsally and the osteoblast surface ventrally. Finite element analysis of mechanical stress on the clivus revealed that compressive and tensile stresses appeared mainly on respective dorsal and ventral surfaces of the basioccipital bone. Osteoclastic bone resorption occurred primarily in the compression area, whereas areas of bone formation largely coincided with the tension area. These data collectively suggest that compressive and tensile stresses govern respective localization of osteoclasts and osteoblasts. Developmental analysis of the basioccipital bone revealed the clivus to be angled in early postnatal wild-type mice, whereas its slope was less prominent in *Tnfsf11*^−/−^ mice, which lack osteoclasts. We propose that osteoclast-osteoblast “*trans*-pairing” across cortical bone is primarily induced by mechanical stress from growing organs and regulates shape and size of bones that encase the brain.

## Introduction

Bones provide crucial support for the body and encase organs, such as the brain, spinal cord, lungs and some sense organs. After formation by membranous ossification or endochondral ossification, bones alter their shape and increase in size in response to mechanical stresses by growing organs^1–3^. The two major cell types responsible for reshaping bone morphology are bone-resorbing osteoclasts and bone-forming osteoblasts. Osteoclasts are derived from hematopoietic precursors in response to stimulation by macrophage colony-stimulating factor and RANKL (encoded by *Tnfsf11*) and become tartrate-resistant acid phosphatase (TRAP)-positive multinucleated cells^4,5^. Osteoblast-osteocyte lineage cells differentiate from mesenchymal precursors and produce RANKL. Bone would become too thick and space-occupying or too thin and perforated without a controlled balance between osteoclastic bone resorption on one side and osteoblastic bone formation on the other. However, spatiotemporal regulation of bone resorption and formation activities during bone modelling, especially mechanisms allowing bones that encase organs to undergo morphological changes, remains unclear.

Wolff’s Law, stated in 1892, postulated that bone structurally adapts to mechanical influence. That law was later re-formulated by Frost as bone modelling drift comprised of formation drifts by osteoblasts and resorption drifts by osteoclasts^6^. One example of modelling drifts is illustrated by orthodontic tooth movement^7–9^. After orthodontic force application, osteoclasts resorb the compression side of alveolar bone, towards which the teeth are driven, whereas osteoblasts form bone at the opposite tension side^10^. Long bones also respond to axial loading and increase bone formation, as has been shown in experimental animals^11–13^.

During development, growing organs exert mechanical stress on surrounding bones, and modelling drifts have been observed in diverse contexts. In bone modelling of the neural and hemal arches of the teleost fish medaka, osteoclasts exist on the inner side of the arch and osteoblasts on the outer, expanding arch diameter to allow passage of the spinal cord or blood vessels^14^. Indeed, bone resorption plays a primary role in enlargement of curved skeletal elements such as skull bones during teleost development^15^. In mammals, the calvaria and cranial base grow in parallel with rapid brain expansion, which occurs in the first 2 years in humans, 2 months in rats, and 20 days in mice^16,17^. In mice, intracranial pressure or volume, as well as skull length, sharply increase between postnatal day 0 (P0) and P20^18,19^. The calvaria ossifies by membranous ossification, and open sutures such as the metopic, coronal, sagittal and lambdoid allow the cranium to grow synchronously with underlying brain^20^. Histological analysis in mice shows that TRAP-positive osteoclasts spread over the endocranial surface of the calvarial bone to enlarge the brain cavity^21^. The cranial base, including the basioccipital bone, which ossifies by endochondral ossification, is widely thought to be central to growth and patterning of the skull^22^. Indeed, changes in basicranial growth at synchondroses modulate calvarial and facial shape in mice and humans^23–25^.

The dorsal surface of the basioccipital bone gradually forms a sloping surface called the clivus, which extends from the dorsum sellae to the foramen magnum. Using clivus development as a model, we tested the hypothesis that mechanical force induced by expansion of adjacent organs governs osteoclast and osteoblast distribution during development. To this end, we analysed localization of bone cells in basioccipital bone. We also performed finite element analysis (FEA) to evaluate whether mechanical force accounted for osteoclast localization on the endocranial (cerebral) surface and osteoblast localization on the ectocranial (nasopharyngeal) surface of the basioccipital bone.

## Methods

### Mice

*Col1a1*-AcGFP transgenic mice, which express *Aequorea coerulescens* green fluorescent protein in osteoblasts, were established as previously described^26^. *Tnfsf11*^−/−^ mice were on a C57BL/6 J genetic background^27^. Wild-type (WT) and heterozygous littermates served as controls. Mice were decapitated under anaesthesia with sevoflurane. After removing skin, heads were fixed overnight in 2% paraformaldehyde (PFA) in PBS in order to prepare frozen sections, or in 4% PFA in PBS to prepare paraffin sections. Where indicated, WT mice (CLEA Japan) were injected with anti-RANKL monoclonal antibody (5 μg/g body weight, 47104900, ORIENTAL YEAST) at P1 and alizarin complexone (as described below) at P3, and were sacrificed at P4. All mice were housed in a specific pathogen-free barrier facility and maintained on a 12-hour light/dark cycle. Animal experiments were carried out in accordance with Institutional Guidelines on Animal Experimentation at Keio University, and protocols were approved by the Keio University Institutional Animal Care and Use Committee.

### Safranin O staining

Synchondroses in paraffin sections made from the demineralized cranial base were stained with safranin O. Briefly, 4% PFA/PBS-fixed mouse heads were decalcified in 10% EDTA/0.1 M Tris (final pH 7.0) for one week at room temperature with agitation and embedded in paraffin. Paraffin sections (4 μm) were cut with a microtome blade (PATH BLADE + PRO, Matsunami) on a sliding microtome (Yamato Kohki, REM-710). After deparaffinization, sections were stained with Weigert’s iron haematoxylin solution, washed with 0.5% HCl in 70% ethanol, and stained sequentially in 0.05% fast green (Polysciences) and 0.1% safranin O (Polysciences). Slides were then evaluated by microscopy (BX53, Olympus).

### Tartrate-resistant acid phosphatase (TRAP) staining

Paraffin sections were stained for TRAP activity using an Acid Phosphatase, Leukocyte (TRAP) kit (Sigma-Aldrich, 387 A) to detect osteoclasts. Sections were incubated 10 minutes at 37 °C, and the reaction was stopped by washing in distilled water. Whole-mount TRAP staining of basioccipital bones was similarly performed after fixation in 4% PFA/PBS. TRAP-positive areas (%) were measured after binarization of staining intensities. Frozen sections were stained for TRAP activity using ELF 97 (Thermo Fisher Scientific) as substrate.

### Immunohistochemistry

Immunostaining was conducted on 6 or 10 μm frozen cryostat sections of the undecalcified head, according to the method of Kawamoto (Leica, CM3050S)^28^. Sections were blocked with 1% BSA/PBS and then incubated with goat anti-mouse MMP-9 polyclonal antibody (AF909, R&D) or rabbit anti-mouse collagen type I polyclonal antibody (AB765P, Merck Millipore) overnight at 4 °C, followed by donkey anti-goat Alexa Fluor 647 or donkey anti-rabbit Alexa Fluor 488 secondary antibodies (Thermo Fisher Scientific), respectively. Osteocalcin was detected on demineralized paraffin sections that had been deparaffinized, treated with 1% H_2_O_2_ in methanol, blocked with 1% BSA/PBS, and incubated with rabbit anti-osteocalcin polyclonal antibody (ALX-210-333, Enzo). Corresponding nonimmune rabbit IgG served as a control. Horse anti-rabbit IgG-HRP secondary antibody (MP-7401, VECTOR) was detected with a diaminobenzidine (DAB) HRP substrate (VECTOR). Sections were observed under a confocal laser microscope (LSM-710, Zeiss; FV3000, Olympus) or a light microscope (BX53, Olympus).

### Bone labelling

Alizarin complexone (Sigma-Aldrich) (3 mg/ml in 2% NaHCO_3_) was injected subcutaneously into mice at 10 μl/g body weight. Calcein (1.6 mg/ml in 2% NaHCO_3_) was injected into mice at 10 μl/g body weight. Labelled bones were prepared for frozen sections according to the method of Kawamoto (Leica, CM3050S)^28^.

### Micro-computed tomography (Micro-CT)

Samples were fixed in 2 or 4% PFA in PBS overnight. CT images were obtained using a micro-CT apparatus (R_mCT2, Rigaku) under 90 kV, 160 μA and 512 projections/360°. Fields of view were 30 × 30 mm (isotropic voxel size, 60 μm), 20 × 20 mm (40 μm), and 5 × 5 mm (10 μm). Contrast staining was performed using 3.75% Lugol’s solution (I_2_KI) in 70% ethanol^29^. A bone mineral density (BMD) colour map was obtained using TRI/3D-BON software (Ratoc System Engineering). Bone length and thickness were measured using Image J (NIH).

### Finite Element Analysis (FEA)

FEA models were generated by directly converting micro-CT voxels (cubes) to elements using bone analysis software (TRI/3D-BON, Ratoc System Engineering). In this model, the basioccipital bone was fixed on the ventral side at three different synchondroses (the spheno-occipital synchondrosis and basioccipital-exoccipital synchondroses) and was sandwiched dorsally with a soft tissue model of the brainstem (elastic modulus: 1 kPa, Poisson’s ratio: 0.48) and ventrally with a soft tissue model of a muscle (elastic modulus: 12 kPa, Poisson’s ratio: 0.45)^30^ (Supplementary Fig. S1). Elastic modulus of bone^31^ in the TRI/3D-Bon software was given by E(Pa) = 16.311 × [BMD(mg/cm^3^)]^3^ (Poisson’s ratio: 0.3) according to the manufacturer. Vertical load of 0.005 N was applied from the dorsal surface to simulate intracranial pressure and brain mass. Compressive stress (minimum principal stress) and tensile stress (maximum principal stress) were analysed.

### Statistical analysis

Data are expressed as means ± standard deviation (SD) and were analysed by Student’s t-test.

## Results

### Localization of osteoclasts and osteoblasts in basioccipital bone

**Figure 1:**
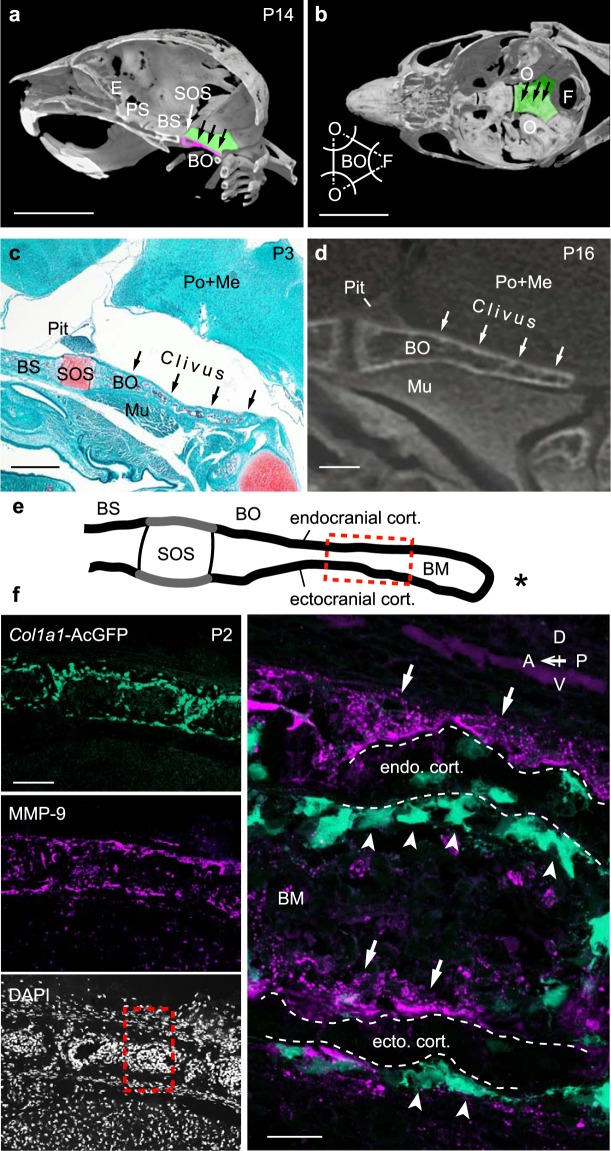
Localization of osteoclasts and osteoblasts in the basioccipital bone. (**a**) Representative micro-CT image of a mouse skull at P14 (n = 4). E, ethmoid; PS, presphenoid; BS, basisphenoid; BO, basioccipital (dorsal surface, green; parasagittal section, magenta); SOS, spheno-occipital synchondrosis; Arrows indicate the clivus. Voxel size, 40 μm. Scale bar, 5 mm. (**b**) Dorsal view of the cranial base shown in (**a**) and a scheme of the basioccipital bone. O, otic capsule; F, foramen magnum. Arrows indicate the clivus. Scale bar, 5 mm. (**c**) Safranin O staining of the cranial base in a representative section from a P3 mouse (n = 6). Arrows indicate the clivus of the basioccipital between the brainstem (pons and medulla, Po + Me) and longus capitis muscle (Mu). Pit, pituitary; Scale bar, 500 μm. (**d**) I_2_KI-contrast staining in a representative sample from a P16 mouse (n = 1). Arrows indicate the clivus. Voxel size, 10 μm. Scale bar, 500 μm. (**e**) Schematic showing the cranial base at an early postnatal stage. Asterisk, foramen magnum. BM, bone marrow. Red boxed region indicates area shown in (**f**). (**f**) *Col1a1*-AcGFP and MMP-9 immunostaining in representative sections from a P2 mouse (n = 2). (Left) Fluorescence detection of AcGFP (green), MMP-9 (magenta), and DAPI staining (white) in a P2 mouse. Scale bar, 100 μm. (Right) Magnified view of the boxed area in “DAPI”. Broken lines indicate endocranial and ectocranial cortical bone layers. Arrows, osteoclasts; arrowheads, osteoblasts; BM, bone marrow between cortical bones. A, anterior; P, posterior; D, dorsal; V, ventral. Maximum intensity projection images were acquired using a 63x objective and assembled from 3 slices at 2.86 μm intervals. Scale bar, 20 μm.

To examine morphology of the murine clivus, we acquired micro-CT images of the skull of P14 mice. Analysis of a parasagittal view showed that the cranial base consisted of the ethmoid, presphenoid, basisphenoid, and basioccipital bones (Fig. 1a). At this stage, the osseous clivus is seen sloping downward to the foramen magnum, an opening that allows passage of the spinal cord. A dorsal view of the clivus is shown in Fig. 1b. In this case, the basioccipital bone resembles an equilateral triangle on the midline, with each vertex removed by an arc of a circle. One posterior circle coincides with the foramen magnum (Fig. 1b, F) and the two anterolateral circles with the otic capsules (Fig. 1b, O).

We next visualized tissues surrounding the clivus of basioccipital bone in P3 mice in sagittal sections stained with Safranin O, which stains cartilage red. The Safranin O-positive spheno-occipital synchondrosis (Fig. 1c, SOS) was ventral to the pituitary (Fig. 1c, Pit), and the basioccipital bone, which gives rise to the clivus (arrows in Fig. 1c), was below the brainstem (pons and medulla) but above the longus capitis muscle (Fig. 1d, Mu). Since the brainstem is only loosely attached to the basioccipital bone, evaluation of their spatial relationship in histological sections was challenging. As an alternative, we performed micro-CT imaging after I_2_KI-contrast staining of the P16 murine head. The basioccipital consisted of endocranial (dorsal) and ectocranial (ventral) cortical bone layers with a bone marrow space between them, and the basioccipital was sandwiched between the brainstem and the longus capitis muscle (Fig. 1d). These data strongly suggest that the clivus is compressed dorsally by the brainstem during brain enlargement.

To localize osteoblasts and osteoclasts in postnatal basioccipital bone (Fig. 1e), we visualized osteoblasts using *Col1a1*-AcGFP transgenic P2 mice and detected osteoclasts by MMP-9 immunostaining. Strikingly, AcGFP-positive osteoblasts were localized to the ventral sides of both endocranial and ectocranial cortical bone layers, towards the foramen magnum. Similarly, MMP-9-positive osteoclasts were seen on the dorsal surfaces of both cortical bone layers (Fig. 1f). These data indicate that osteoclast and osteoblast surfaces are paired on the opposite sides of each cortical bone layer of the developing basioccipital bone. In contrast to bone remodelling, which occurs on the same surface of bone in *cis*, we designate the paired distribution of osteoclast and osteoblast surfaces across cortical bone as “*trans*-pairing” between osteoclasts and osteoblasts.

### Compression and tension surfaces correspond to respective osteoclast and osteoblast surfaces

**Figure 2:**
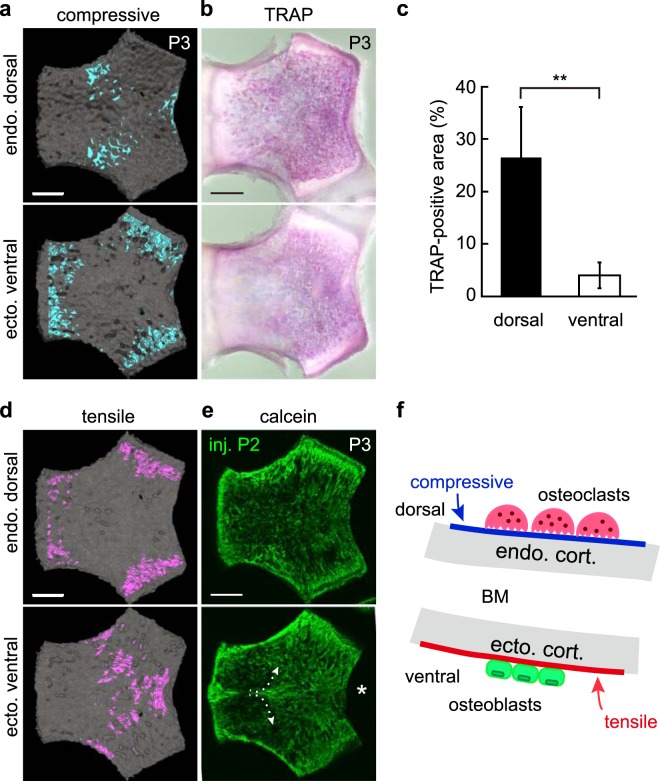
Finite element analysis (FEA) of the relationship between mechanical stresses and bone resorption or formation surfaces. (**a**) Micro-CT-based FEA of basioccipital bone surfaces at P3 (n = 1). Blue indicates area of compressive stress from −0.096 to -0.98 MPa. (**b**) Whole-mount TRAP staining of the basioccipital bone in P3 mice (n = 5) showing localization of TRAP activity. (**c**) Graph shows TRAP-positive area of dorsal surface (26.5 ± 4.3%) and ventral surface (4.1 ± 1.1%, **p = 0.0053, n = 5). (**d**) FEA of basioccipital bone surfaces at P3. Red indicates area of tensile stress from 0.077 to 0.60 MPa. (**e**) Calcein bone labelling (injected at P2) in P3 mice (n = 3). Arrows indicate bone formed in a triangular area on the ventral surface, starting at the midline and spreading towards the foramen magnum (asterisk). (**f**) Schematic showing *trans*-pairing in postnatal clivus development and illustrating osteoclastic bone resorption on the compression surface (blue line) and osteoblastic bone formation on the tension surface (red line). All scale bars, 500 μm.

We asked whether mechanical stress is crucial for formation of osteoclast and osteoblast surfaces in the basioccipital bone by performing finite element analysis (FEA), an *in silico* method that predicts stress or strain distribution in response to mechanical loading. To do so, we constructed a FEA model based on voxels of a micro-CT image of P3 mice, and loading was applied to the entire dorsal surface of a basioccipital bone model through brain tissue (Supplementary Fig. S1). As a result, we detected compressive stress on the dorsal surface of the basioccipital bone (Fig. 2a, top), while almost no signal was seen on the ventral surface except for the fixed areas for FEA, namely, the three synchondroses junctions (Fig. 2a, bottom). To evaluate areas of bone resorption, we performed whole-mount TRAP staining in P3 mice (Fig. 2b). A larger percentage of the total area was TRAP-positive on the dorsal versus the ventral side of the basioccipital bone, reflective of greater osteoclast activity in the former (Fig. 2c). These data suggest that TRAP-positive areas, which correspond to resorbed areas, largely correspond to compression areas in FEA (Fig. 2a–c).

By contrast, we detected tensile stress on the ventral surface of the basioccipital bone, with almost no signal on the dorsal surface except for the fixed areas for FEA (Fig. 2d). Calcein bone labelling analysed in P3 mice indicated bone formation occurring in a triangular area on the ventral surface (Fig. 2e, arrows), corresponding to the tensile area in FEA. These data suggest that mechanical stresses govern osteoclast and osteoblast localization across cortical bone during postnatal clivus development (Fig. 2f).

### Osteopetrotic mice exhibit misalignment of osteoblasts

**Figure 3:**
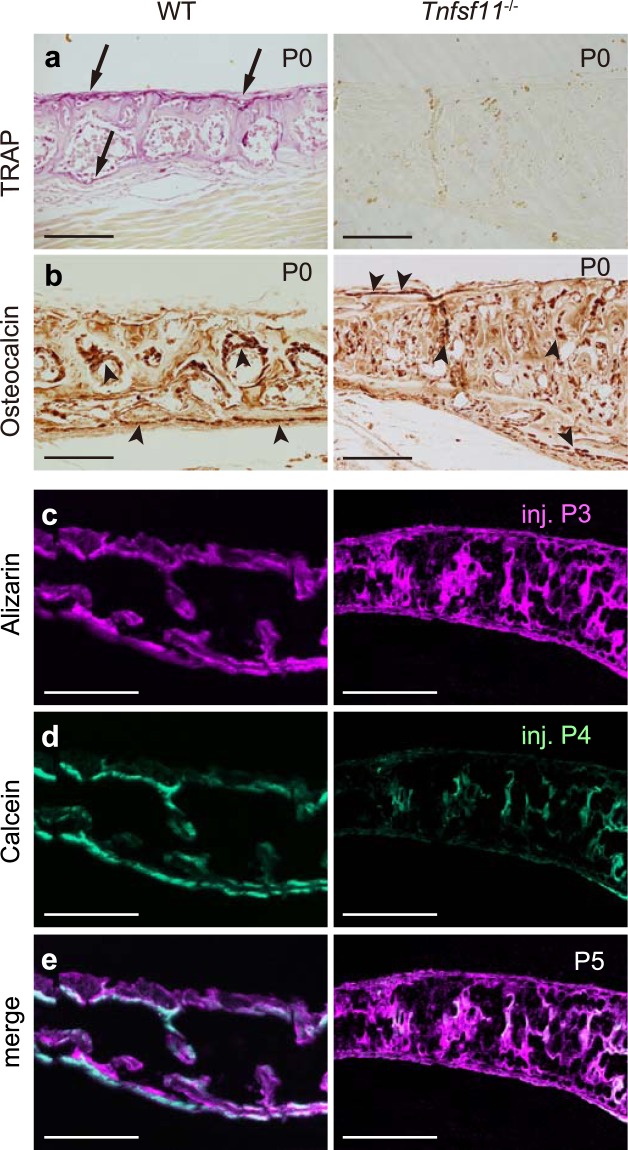
Misalignment of osteoblasts in *Tnfsf11*^−/−^ mice. Areas shown in all panels correspond to the boxed area in the basioccipital bone in Fig. 1e. (**a**) TRAP staining in representative sections of WT (n = 3) and *Tnfsf11*^−/−^ (n = 2) mice at P0. Arrows, TRAP-positive osteoclasts at the dorsal periosteum and endosteum. Scale bars, 100 μm. (**b**) Osteocalcin-positive osteoblasts (arrowheads) in representative sections of WT and *Tnfsf11*^−/−^ mice at P0. Scale bars, 100 μm. (**c**–**e**) Alizarin complexone (**c**, injected at P3) and calcein (**d**, injected at P4) double-labelling (**e**, merged image) in representative sections of WT (n = 3) and *Tnfsf11*^−/−^ (n = 2) mice at P5. Scale bars, 200 μm.

To determine whether osteoblast localization changes in the absence of osteoclasts, we first analysed osteopetrotic RANKL-deficient (*Tnfsf11*^−/−^) mice, which lack osteoclasts. Wild-type (WT) mice at P0 showed TRAP-positive osteoclasts on the dorsal surface of each cortical bone layer (Fig. 3a, arrows), and TRAP activity was particularly robust immediately underneath the dorsal periosteal dura. As expected, we observed no TRAP-positive cells in *Tnfsf11*^−/−^ mice at P0 (Fig. 3a, right). In WT mice at P0, osteocalcin-positive osteoblasts were aligned on the ventral surfaces of ectocranial and endocranial cortical bone layers (Fig. 3b, left). In *Tnfsf11*^−/−^ mice at P0, osteocalcin-positive osteoblasts were hardly detectable and not aligned ventrally, suggesting that osteoclasts control ventral surface localization of osteoblasts (Fig. 3b, right). Next, to visualize the bone formation area in the basioccipital, we subcutaneously injected WT or *Tnfsf11*^−/−^ mice with two fluorochromes, alizarin complexone at P3 and calcein at P4, and harvested the cranial base at P5. In WT mice, we observed calcein labelling at the ventral surfaces of both endocranial and ectocranial cortical bone layers, indicating deposition of bone matrix on the ventral surfaces (Fig. 3c–e left). By contrast, we observed only low levels of calcein labelling in *Tnfsf11*^−/−^ mice and no strong ventral-specific bone formation in the absence of osteoclasts (Fig. 3c–e, right). These data suggest that the osteoblast-surface does not form properly in the absence of osteoclasts.

**Figure 4:**
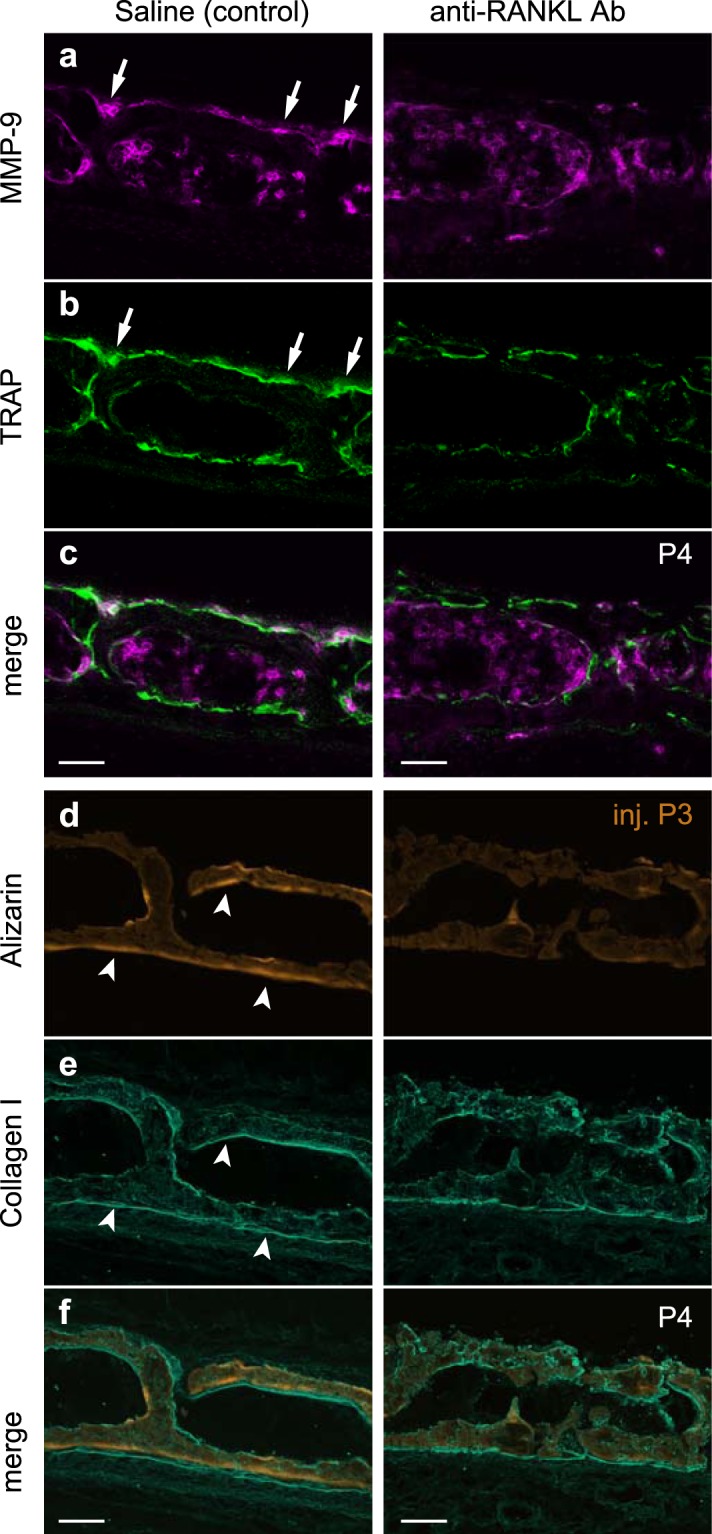
Mice injected with anti-RANKL antibody. Mice were injected with saline or anti-RANKL antibody at P1 and then injected with alizarin complexone at P3. The basioccipital bone was then analysed at P4 (n = 4 each group). (**a**) MMP-9 immunostaining. (**b**) TRAP activity staining. (**c**) Merge image of MMP-9 and TRAP staining. (**d**) Alizarin labelling. (**e**) Collagen type I immunostaining. (**f**) Merged image of alizarin labelling and collagen type I staining. Bone resorption (arrows) and formation (arrowheads) surfaces are indicated. Maximum intensity projection images were acquired using a 40x objective and assembled from 12 slices at 0.6 μm intervals. Scale bars, 50 μm.

Osteopetrotic mice exhibit abnormal bone structure and impaired endochondral ossification, making detailed analysis of resultant phenotypes difficult. To overcome this limitation and perturb osteoclastic bone resorption, we injected saline or anti-RANKL antibody at P1 and alizarin complexone at P3 in WT mice. As expected, in saline-injected control mice, we detected MMP-9-positive osteoclasts on the dorsal surfaces of cortical bone layers at P4 (Fig. 4a, arrows). By contrast, we detected fewer MMP-9-positive osteoclasts on the dorsal surface in anti-RANKL antibody-injected mice (Fig. 4a, right). MMP-9-positive cells on the dorsal surfaces, but not those in bone marrow, were further confirmed to be TRAP-positive osteoclasts (Fig. 4b,c). Strikingly in anti-RANKL antibody-injected mice, bone formation at the ventral surfaces was dramatically reduced based on lack of alizarin labelling (mineralized bone, Fig. 4d) and less prominent collagen type I staining (osteoid) compared with saline -injected controls (Fig. 4e,f). These data suggest that active bone formation on the ventral surface depends on the presence of dorsal osteoclasts.

### Clivus development requires osteoclasts and *trans*-pairing

**Figure 5:**
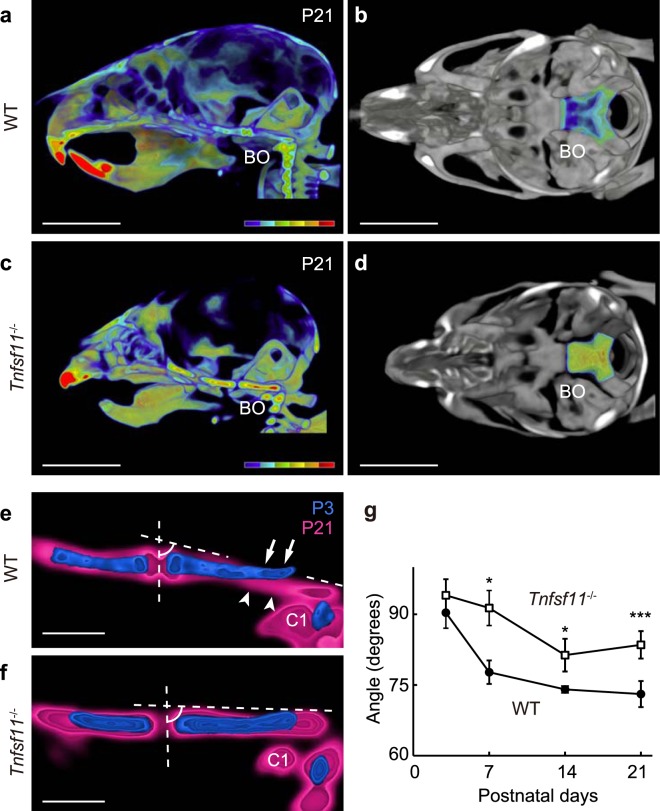
Clivus development requires osteoclast-osteoblast *trans*-pairing. (**a**) Midsagittal plane of a representative micro-CT image of WT mouse skull at P21. Colour bar shows bone mineral density (BMD) levels, from 1 (blue) to 600 (red) mg/cm^3^. BO, basioccipital bone. Scale bar, 5 mm. Voxel size, 60 μm. (**b**) Dorsal view of the WT cranial base. Scale bar, 5 mm. (**c**) Midsagittal plane of a representative micro-CT image from a *Tnfsf11*^−/−^ mouse at P21 (n = 6). Colour bar, as in (**a**). Scale bar, 5 mm. (**d**) Dorsal view of sample in (**c**). BMD of BO is higher than that of WT BO. Scale bar, 5 mm. (**e**) Comparison of basioccipital bones at P3 (blue) and P21 (magenta) in representative micro-CT images from WT mice (P3, n = 4; P21, n = 8). Bone matrix is resorbed on the dorsal side (arrows) and forms on the ventral side (arrowheads) of the basioccipital bone. Dashed lines indicate the clivus angle, as defined by the angle between the chondro-osseous junction (vertical) and the dorsal surface of the basioccipital bone at the midline. The clivus develops towards cervical vertebra 1 (C1). Scale bar, 1 mm. Voxel size, 40 μm (P3) and 60 μm (P21). (**f**) Analysis similar to (**e**) in *Tnfsf11*^−/−^ mice. Clivus formation is not prominent between P3 (blue) and P21 (magenta). Scale bar, 1 mm. (**g**) Postnatal temporal changes in clivus angle in WT (closed circles. P3, n = 4; P7, n = 6; P14, n = 4; P21, n = 8) and *Tnfsf11*^−/−^ (open squares. P3, n = 3; P7, n = 3; P14, n = 4; P21, n = 6) mice. The data are shown as means ± SD. Day 7, *p = 0.011; Day 14, *p = 0.022; Day 21, ***p = 0.00004.

To examine the effect of osteoclast loss on clivus development, we examined skull shape in WT and *Tnfsf11*^−/−^ mice at P21. We first made colour-coded micro-CT images of the skull to evaluate bone mineral density (BMD) (Fig. 5a–d). Relative to WT mice, *Tnfsf11*^−/−^ mice showed a shortened basicranium, round calvaria, and no tooth eruption, all well-characterized phenotypes of osteopetrotic mice (Fig. 5c, Supplementary Fig. S2). BMD of the skull, especially calvaria, was generally lower in *Tnfsf11*^−/−^ than in WT mice at P21 (Fig. 5c), suggesting that loss of osteoclastic bone resorption impairs osteoblastic bone formation. The apparent higher BMD of the cranial base seen in mutant mice is likely due to unresorbed calcified cartilage.

We next compared basioccipital bones in WT and *Tnfsf11*^−/−^ mice in the midsagittal plane by overlaying micro-CT images at P3 and P21 and aligning the spheno-occipital synchondrosis horizontally in the middle (Fig. 5e,f). In WT mice at P21, the endocranial surface of the basioccipital bone appeared to have been resorbed (Fig. 5e, arrows), while bone matrix of the ectocranial surface had formed (Fig. 5e, arrowheads). In the midsagittal plane of WT mice at P21, the posterior end of the basioccipital bone had developed downwards along the dorso-ventral axis towards the first cervical vertebra (C1), forming an angle from the dorsum sellae to the foramen magnum (Fig. 5e). By contrast, in the basioccipital bone of *Tnfsf11*^−/−^ mice, the clivus remained relatively horizontal, and the distance between the basioccipital bone and C1 was greater at P21 than in comparably-aged WT control mice (Fig. 5f). These data show that the slope of the clivus in *Tnfsf11*^−/−^ mice was less than that seen in WT mice.

Finally, we measured the angle between the dorsal surface of the basioccipital and the chondro-osseous junction as indicative of the clivus angle (Fig. 5e,f, dashed lines) in WT and *Tnfsf11*^−/−^ mice at P3, P7, P14 and P21 using micro-CT images. At P3, that angle was comparable in WT and mutant mice. In WT mice, however, the clivus angle rapidly decreased by P7, whereas in *Tnfsf11*^−/−^ mice, that decrease was more gradual, and clivus angles in *Tnfsf11*^−/−^ mice at P7, P14 and P21 were significantly greater than those seen in WT mice (Fig. 5g). These data suggest that clivus development requires osteoclastic bone resorption and *trans*-pairing.

## Discussion

We demonstrate here that paired localization of osteoclast and osteoblast surfaces across cortical bone induces bone drift to enlarge the cranial cavity. During human growth, the basioccipital bone is resorbed on the endocranial surface and forms on the ectocranial surface^32^. The novelty of our study lies in finding that individual cortical layers, endocranial and ectocranial, of the basioccipital bone carry the osteoclast surface dorsally and the osteoblast surface ventrally, and that such osteoclast/osteoblast localization can be explained by mechanical stress. These observations lead us to propose the term, osteoclast-osteoblast “*trans*-pairing,” in which osteoclastic bone resorption occurs on one surface of bone and balanced osteoblastic bone formation on the other.

Micro-CT analysis of the I_2_KI contrast-stained mouse cranial base revealed that the brainstem is localized on top of the endocranial cortical bone, suggesting that the basioccipital bone is exposed to mechanical forces induced by enlargement of these brain structures. We created an FEA model of P3 basioccipital bone from micro-CT images, and loading was applied onto the dorsal surface *in silico* through soft material simulating brain tissue. The theoretical area of compressive stress emerged near the centre of the dorsal surface of the basioccipital bone model, coinciding with the observed distribution of osteoclasts. Furthermore, the theoretical area of tensile stress corresponded positively to the observed area of bone formation, a triangular area spreading towards the foramen magnum on the ventral surface. Therefore, we propose that *trans*-pairing between osteoclasts and osteoblasts is primarily induced by mechanical stress exerted by expanding organs. A limitation of this FEA model is that it does not consider potential effects by radial expansion of the spinal cord and various other micro-motions. More sophisticated FEA would improve the simulation. In addition, more realistic boundary conditions, namely the loads and constraints, should be evaluated.

Assuming that FEA accurately captured osteoclast and osteoblast surfaces, one might wonder which cell type, pre-osteoclasts, pre-osteoblasts, osteocytes or other cells, respond to mechanical stress and mediate interactions between osteoclasts and osteoblasts. Osteocytes buried in bone matrix reportedly sense mechanical stress^33^ and can reciprocally regulate osteoclastic bone resorption and osteoblastic bone formation through regulation of the Wnt antagonist sclerostin (encoded by *Sost*) and RANKL^34^. It is noteworthy, however, that osteocytes *per se* may not be an essential component of osteoclast-osteoblast pairing, as medaka fish, which lack osteocytes, show apparent osteoclast-osteoblast *trans*-pairing^14,35^. In mice, osteoblasts release nerve growth factor in response to mechanical force to activate TrkA-positive sensory nerves, which secrete osteogenic cues to stimulate bone formation^36^. Alternatively, tensile stress might directly activate mesenchymal osteoprogenitors or osteoblasts. It is worth noting that tensile force-responsive osteogenesis of alveolar bone is mediated by bone morphogenetic protein (BMP) signalling^37^.

On the dorsal surfaces of the basioccipital bone, periosteal dura might primarily respond to compressive stress to activate osteoclasts. It is plausible that compression of the periosteal dura on the dorsal surface of the basioccipital bone upregulates RANKL production and promotes osteoclast differentiation, as is the case for periodontal ligament (PDL) cells^7,38^. Dural cerebral veins^39^ are the likely source of osteoclast precursors. Curiously, in the basioccipital bone we observed that when the number of osteoclasts was experimentally decreased by injecting mouse pups with anti-RANKL antibody, osteoblast activity on the other side of cortical bone also decreased. In other words, osteoclasts may activate osteoblasts on the other side of bone through osteoclast-derived osteogenic factors similar to so-called coupling factors^1,40–44^. In addition to activation, maintenance of a quiescent surface by osteoclast-derived factors may be required for proper localization of osteoblasts at a certain distance from osteoclasts^45^. Identification of relevant molecules or exosomes, which act at a distance across cortical bone, awaits future studies.

We previously reported that osteopetrotic mice, including *Tnfsf11*^−/−^ mice, show hearing loss due to narrowing of the middle ear cavity that impedes vibration of auditory ossicles^46^. This phenotype can be seen as a consequence of impaired *trans*-pairing associated with impaired endochondral ossification. To determine whether a lack of osteoclasts alters generation of the clivus downward slope, we measured clivus angles between the vertical line and dorsal surface of the basioccipital bone. In WT mice, those angles became progressively less than 90 degrees by P7, contributing to formation of a funnel-like opening of the foramen magnum. Temporally, clivus development occurs during a period of rapid growth of the brain, which is facing the cranial base^47^. In osteopetrotic *Tnfsf11*^−/−^ mice, clivus development was impaired, supporting our view that osteoclast-osteoblast pairing is essential to reshape the basioccipital bone.

In conclusion, during postnatal reshaping of the basioccipital bone, mechanical stress applied by the enlarging brain likely induces osteoclast-osteoblast *trans*-pairing characterized by paired localization of osteoclast and osteoblast surfaces across each cortical layer. Our results provide insight into mechanisms that coordinate growth of bone and encased organs throughout the body.

## Supplementary information

Supplementary Figures S1 and S2

## Data Availability

No large datasets were generated or analysed during this study. All other datasets analysed here are available from the corresponding author on reasonable request.
