# Supplementary material for: Trans-pairing between osteoclasts and osteoblasts shapes the cranial base during development
Source: Sci Rep. 2019 Feb 13;9:1956. doi: 10.1038/s41598-018-38471-w (PMC6374512; doi:10.1038/s41598-018-38471-w)
Supplement: Supplementary file 1 — Supplementary Figures S1 and S2 [file 41598_2018_38471_MOESM1_ESM.pdf]

Trans-pairing between osteoclasts and osteoblasts shapes the cranial base during development

Mio Edamoto, Yukiko Kuroda, Masaki Yoda, Katsuhiro Kawaai & Koichi Matsuo

Supplementary Figure 1

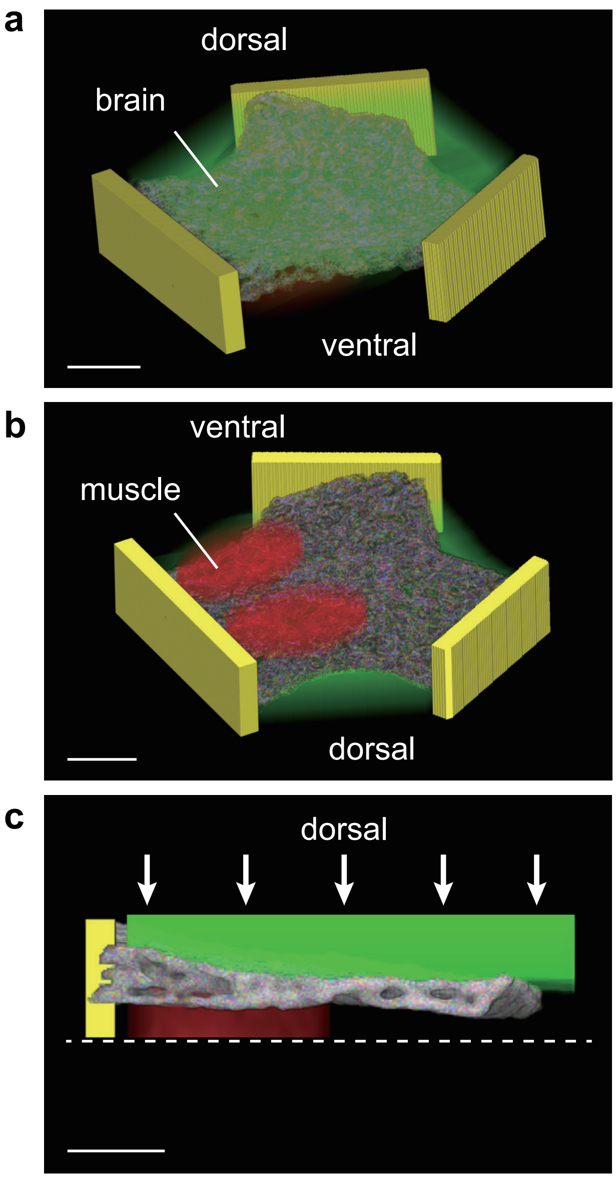

Supplementary Figure S1. Finite element (FE) model. (**a**) A dorsal oblique view from the left side of the FE model created based on a CT image of a mouse skull at P3. The basioccipital bone (grey) was fixed at the three synchondroses with three solid supports (yellow). Soft tissues simulating the brain (green) and muscle (red) were placed on the dorsal and ventral sides, respectively. (**b**) A ventral oblique view. (**c**) Midsagittal section. Uniformly distributed loading was applied dorsally (arrows). The model is supported by a solid plane (dotted line). Scale bars, 500 μm.

Supplementary Figure 2

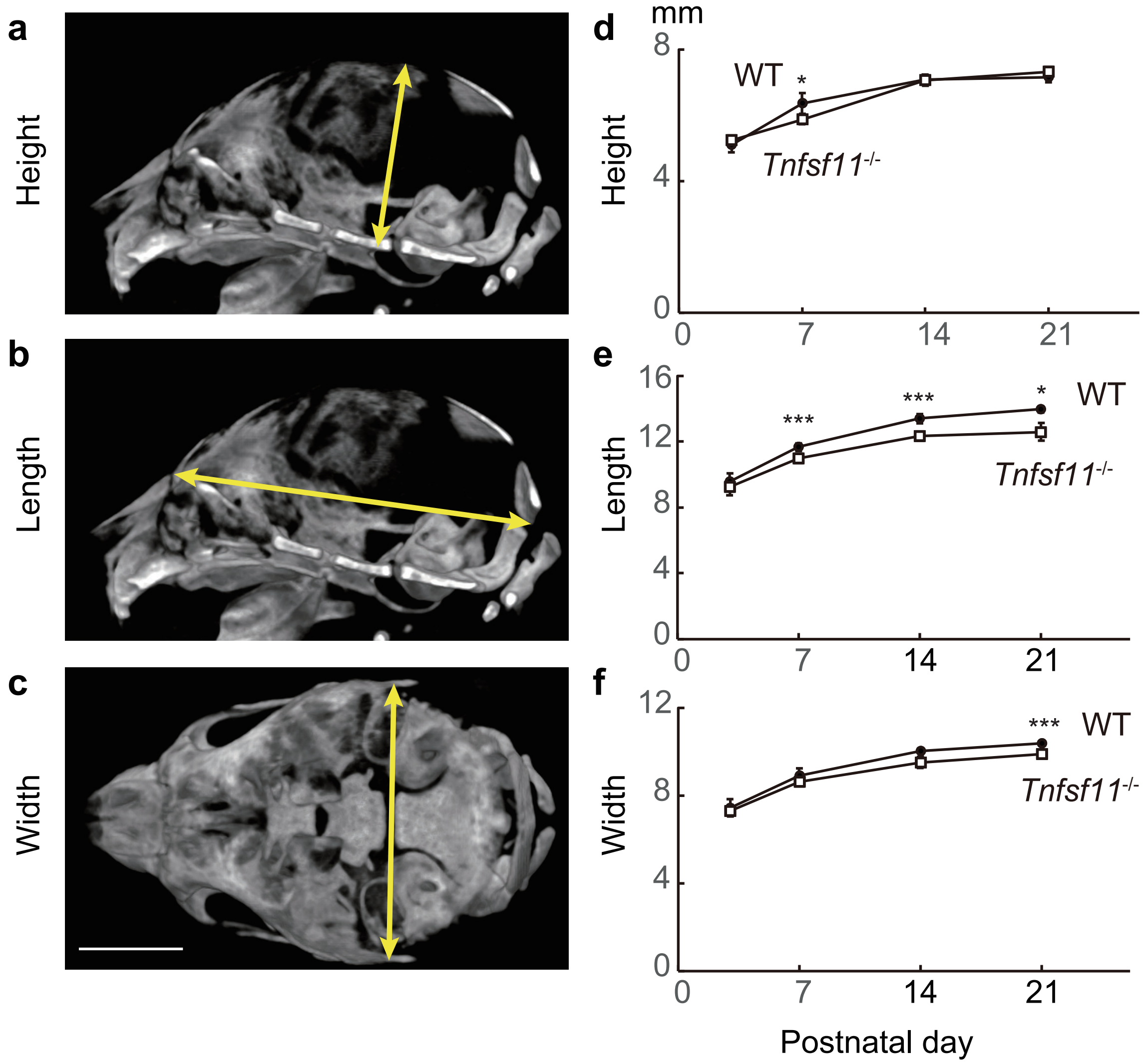

Supplementary Figure S2. Postnatal development of wild-type (WT) and *Tnfsf11*$^{-/-}$ mouse skull.

(**a**-**c**) Micro-CT images showing the height (**a**), length (**b**) and width (**c**) of a P3 WT mouse skull (arrows). (**d**-**f**) Height (**d**), length (**e**) and width (**f**) of developing skulls at P3 (WT, n=4; *Tnfsf11*$^{-/-}$, n=3), P7 (WT, n=6; *Tnfsf11*$^{-/-}$, n=3), P14 (WT, n=4; *Tnfsf11*$^{-/-}$, n=3), and P21 (WT, n=5; *Tnfsf11*$^{-/-}$, n=4). The data are shown as means ± SDs. *p<0.05, **p<0.01, ***p<0.001 vs. WT controls. Statistical analysis was performed with Student's t-test. Note that the skull becomes slightly but significantly longer in WT versus mutant mice after P7. Scale bar, 3 mm. Landmark definitions to measure distance were based on Marghoub *et al* (2018, J Anatomy, 32, 440-448).
